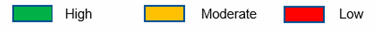# Antimicrobial Stewardship Program: Reducing Antibiotic’s Spectrum of Activity Is not the Solution to Limit the Emergence of Multidrug-Resistant Bacteria

**DOI:** 10.3390/antibiotics11010070

**Published:** 2022-01-07

**Authors:** Rindala Saliba, Assaf Mizrahi, Péan de Ponfilly Gauthier, Le Monnier Alban, Jean-Ralph Zahar, Benoît Pilmis

**Affiliations:** 1 Infection Control Unit, French-Muslim Hospital, Avicenna, 93000 Bobigny, France; saliba.rindala@gmail.com; 2 Clinical Microbiology Department, Hotel-Dieu de France Hospital, Beirut 166830, Lebanon; 3 Service de Microbiologie Clinique, Groupe Hospitalier Paris Saint-Joseph, 75014 Paris, France; amizrahi@ghpsj.fr (A.M.); gpeandeponfilly@ghpsj.fr (P.d.P.G.); alemonnier@ghpsj.fr (L.M.A.); 4 Institut Micalis, Unité Mixte de Recherche 1319, Université Paris-Saclay, INRAe, AgroParisTech, 92290 Châtenay Malabery, France; bpilmis@gmail.com; 5 Equipe Mobile de Microbiologie Clinique, Groupe Hospitalier Paris Saint-Joseph, 75014 Paris, France

**Keywords:** antimicrobial stewardship, dysbiosis, microbiota, de-escalation

## Abstract

Overconsumption of antibiotics in hospitals has led to policy implementation, including the control of antibiotic prescriptions. The impact of these policies on the evolution of antimicrobial resistance remains uncertain. In this work, we review the possible limits of such policies and focus on the need for a more efficient approach. Establishing a causal relationship between the introduction of new antibiotics and the emergence of new resistance mechanisms is difficult. Several studies have demonstrated that many resistance mechanisms existed before the discovery of antibiotics. Overconsumption of antibiotics has worsened the phenomenon of resistance. Antibiotics are responsible for intestinal dysbiosis, which is suspected of being the source of bacterial resistance. The complexity of the intestinal microbiota composition, the impact of the pharmacokinetic properties of antibiotics, and the multiplicity of other factors involved in the acquisition and emergence of multidrug-resistant organisms, lead us to think that de-escalation, in the absence of studies proving its effectiveness, is not the solution to limiting the spread of multidrug-resistant organisms. More studies are needed to clarify the ecological risk caused by different antibiotic classes. In the meantime, we need to concentrate our efforts on limiting antibiotic prescriptions to patients who really need it, and work on reducing the duration of these treatments.

## 1. Introduction

The spread of antimicrobial resistance in hospital and community settings has raised awareness in the medical and scientific world of the need to fight this phenomenon. Although resistance is not exclusive to the human and hospital world, the overconsumption of antibiotics in hospitals and the observation of too many avoidable antibiotic prescriptions have led to the implementation of various control policies. These policies include the control of antibiotic prescriptions to limit or better still, to reduce antibiotic resistance.

These policies have been diverse, ranging from a detailed analysis of national, regional and rarely available local epidemiological data, the introduction of new microbiological diagnostic methods and the recruitment of antibiotic specialists, to policies to restrict the use of antibiotics supposed to have an important ecological impact. One of the essential and major measures introduced in the last two decades is the concept of de-escalation [1,2,3]. This concept refers to a set of measures that aim to reduce the colonization pressure on intestinal microbiota, with the main goal being to reduce the acquisition of multi-drug resistant organisms (MDRO) while maintaining a similar efficacy. With the spread of extended-spectrum beta-lactamase-producing *Enterobacterales* (ESBL-PE), this policy has been intensified and it is characterized by a willingness to spare certain classes of antibiotics (e.g., carbapenems) in favor of other classes of antibiotics that have a narrower spectrum, and therefore less ecological consequences [4,5]. Although several authors have tried to rank the different antibiotic classes based on the concept of “narrow or broad-spectrum”, this classification remains controversial [6] as authors seem to confuse the spectrum of activity and the ecological effect of the different antibiotic classes. Recently, with the deepening of our knowledge of the intestinal microbiota and our better understanding of the factors that participate in the mechanism of resistance to colonization [7], authors have challenged this concept by arguing that different classes of antibiotics could have the same ecological effects [8,9,10]. 

Moreover, despite the introduction of these policies in healthcare settings, their impact on the evolution of antimicrobial resistance remains uncertain. Therefore, it seems important to review the possible limits of such policies and to focus on adopting a better approach, which would consist of adjusting antibiotic prescriptions to be more efficient, and even more so, working on reducing these prescriptions. In this work, we review the literature to address the ecological effect of antibiotics, and attempt to explain why we believe that all antibiotic classes have the same impact on the spread of resistance. We also emphasize the importance of reducing antibiotic prescriptions in the context of limiting the spread of MDRO in healthcare settings.

## 2. Antibiotic Resistance Is an Ancient Phenomenon Enhanced by Antibiotic Prescriptions

Many authors place the emergence of the first resistant bacteria as around the 1940s, right after the discovery of penicillin in 1928 and the onset of its human consumption. It is also common to describe a timeline suggesting a direct link between the discovery of new classes of antibiotics and the emergence of resistance to each of these antibiotics. If there is a temporal correlation between the introduction of new antibiotics and the emergence of new resistance mechanisms to these same antibiotics in clinical practice, it is important to highlight the fact that establishing such a causal relationship is difficult. In fact, if a causal relationship does exist, the cessation or reduction in the consumption of a given antibiotic should result in the disappearance of resistance to the same antibiotic. Moreover, it should enable the restoration of the dysbiosis observed in the affected bacterial populations. However, this idea remains exceptional in view of the observations made over more than a decade [11].

The inability of a bacterial population to regain its normal state of sensitivity can be explained by the fact that antibiotics may not be the cause of resistance, but rather that resistance may have preceded their use. Indeed, it is now well demonstrated by studies conducted on permafrost [12,13] that many resistance mechanisms existed before the discovery of antibiotics. For example, D’Costa et al. showed the presence of encoding resistance genes to -lactams, tetracyclines, and glycopeptides in 30,000-year-old permafrost metagenome samples that also contained DNA belonging to mammoths and other animal species [14]. Similar results were also demonstrated by other authors [15,16].

The reservoir of resistance is in the environment. Indeed, it is important to remember that most antibiotics used in medicine or agriculture are derived from bacteria living in the soil such as *Actinomycetes* or *Paenibacillus* [17]. These antibiotics include, for example, streptomycin, tetracycline, chloramphenicol, erythromycin and vancomycin. On the other hand, different microbial species share the same environmental ecological niche. It is therefore obvious that to survive in the same ecological niche, some microorganisms need to produce metabolites capable of inhibiting the toxic activity of antibiotics produced by other microorganisms; hence, the emergence of resistance mechanisms. These antibiotic-producing organisms may be the original source of many antibiotic resistance genes that we currently observe in clinical practice, in both humans and animals [18,19]. Indeed, most *Streptomyces* are resistant to an average of 7 to 8 antibiotics, including some that have been recently developed [20]. Moreover, the emergence of multiple antibiotic resistance has been explained, at the bacterial level, by different modes of acquisition. Several mechanisms have been described and can be summarized by the acquisition of resistance mechanisms either through the vertical transmission of resistance genes from parental bacteria and/or through horizontal resistance genes transfer between bacteria of the same or different bacterial species. The evolution of resistance over the last 20 years, with the spread of ESBL-PE, demonstrates the important role of the environment in this phenomenon. In fact, it is well demonstrated that the transfer of resistant genes through plasmid mobilization from an environmental bacterial species, *Kluyvera sp*, was responsible for the spread of ESBL-PE on a worldwide scale [21].

Finally, it is important to note that one of the first bacterial strains collected in the United Kingdom in 1915, before any use of antibiotics, already harbored resistance genes to penicillin and erythromycin [22].

## 3. The Effect of Antibiotics on the Microbiota Is a Complex Phenomenon

### 3.1. Gut Microbiota

It is obvious that the overconsumption of antibiotics at the human, animal and environmental level has worsened the phenomenon of resistance. Moreover, it is clear that antibiotic use is responsible for intestinal dysbiosis, which is alleged to be the source of the emergence of bacterial resistance [23,24,25]. At the individual level, the selective pressure caused by antimicrobials on the gut microbiota promotes the carriage of MDRO. This may result from the overgrowth of bacteria with intrinsic or acquired resistance to the administered antibiotic. It may also ensue from the acquisition of exogenous, pandemic MDRO, including ESBL-E and carbapenemase-producing enterobacteria (CPE) through cross-transmission or environmental sources [26,27]. This colonization phenomenon is complex as it involves many direct and indirect factors, constituting a barrier wall, which is also referred to as “colonization resistance” [28].

This ability to resist colonization in the era of antibiotic resistance control has led researchers to focus on the gut microbiota and the impact of antibiotics on it. The effect of antibiotics on intestinal microbiota can be summarized as follows: (1) spatial liberation of the ecological niche; (2) they provide an additional quantity of nutrients; (3) destruction of sensitive bacterial species and emergence of resistant bacteria; and (4) modification of the microbiota’s diversity and richness [29,30,31]. These effects depend on several factors including (1) the initial composition of the microbiota [32]; (2) the concentration levels of antibiotics in the digestive tract [33,34], with variable effects; and (3) the antibiotic’s activity on anaerobic bacteria (Table 1). 

**Figure 1:**
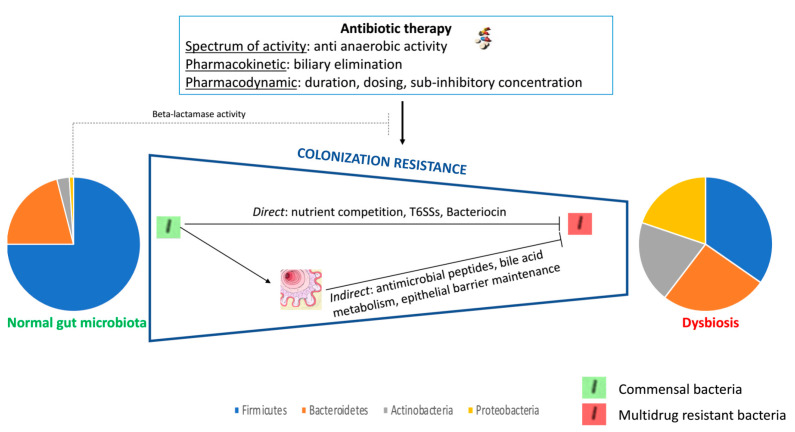
Impact of antibiotic therapy on gut microbiota and colonization resistance.

Although it has several functions, it is important to highlight the role of intestinal microbiota in limiting the acquisition of exogenous pathogenic bacteria and MDRO. While the pharmacokinetics and pharmacodynamics properties of an antibiotic can explain its effect on the intestinal microbiota, it is also important to note that the composition of the microbiota itself plays a role in the acquisition and/or clearance of MDRO. The intestinal microbiota is a complex organ composed not only of bacterial populations, but also of archaea, fungal, parasitic and viral populations. The functions of the microbiota are multiple including the fermentation of food, synthesis of vitamins and amino acids, regulation of the inflammatory/immune system, modulation of gastrointestinal hormone release, regulation of brain behavior, and finally, resistance to colonization by exogenous bacteria. There are many direct and indirect mechanisms involved in resistance to colonization [34,38,43,44,47,48] including bacteriocins, mucus, antimicrobial peptides, nutrient competition, type VI secretion system, and bile acid metabolism (Figure 1). Most of these mechanisms are related to the anaerobic flora. At the bacterial level, the microbiota includes two major phyla, Bacteroidetes and Firmicutes, and two minor phyla, Actinobacteria and Proteobacteria [48]. However, the composition of the microbiota evolves in space and time throughout the digestive tract, and the renewal of species occurs frequently in the same day. Similarly, the distribution of species varies between the lumen of the digestive tract and the surface of its mucosa [49]. If the composition of intestinal microbiota in healthy individuals is identical in different humans, the distribution of bacterial species within the microbiota varies according to many factors, including the type of diet [50]. These differences do not only concern bacterial species but also fungal, parasitic and viral species.

### 3.2. Impact of Antibiotic on Gut Microbiota

The administration of an antibiotic has an effect on different microbiota such as skin, vaginal, respiratory, urinary, and mainly, digestive microbiota. The latter seems to be the most “impacted” because of its richness and diversity (100 trillion bacteria per gram of stool). Numerous longitudinal studies, with varying methodologies, have shown large changes in the intestinal microbiota after the administration of antibiotics [22,51]. These variations, although difficult to compare, suggest changes in the composition of intestinal microbiota (in terms of richness and diversity). They are associated with the emergence of bacterial species that are naturally resistant to the administrated antibiotic and/or species that have acquired resistance mechanisms. All antibiotics have ecological consequences and their effects are long-lasting, up to 24 months after administration for a certain number of molecules [52]. As suggested by Woerther et al., the pharmacokinetic and anti-anaerobic activity of an antibiotic seem to have an important role in the emergence and acquisition of resistant bacteria [53].

### 3.3. Antibiotic Spectrum

The subdivision of available antibiotics into two categories (narrow and broad-spectrum activities) began in the 1980s when cephalosporins were first marketed. Originally, this artificial categorization was used to highlight the “broader” activity of cephalosporins on Enterobacterales, including species that had acquired resistance mechanisms, mainly “penicillinases”. Subsequently, several studies focused on evaluating the impact of each antibiotic class on intestinal microbiota. However, these studies were methodologically questionable and did not compare the impact of one class to another and different molecules within the same class. For many years there seemed to be confusion in regard to the broad spectrum of an antibiotic and its ecological impact. In fact, many authors have attempted to define and classify the ecological effects linked to the administration of different antibiotics [54,55]. However, the proposed classification was based on experts’ opinion and seemed to be influenced by regional epidemiological resistance data [6]. Moreover, as far as we know, despite the large number of studies suggesting the safety of de-escalation [56,57,58], its ecological benefits and effects on intestinal microbiota and on MDRO acquisition have never been demonstrated [6]. More surprisingly, in a recent literature review aiming to identify risk factors associated with the acquisition and emergence of CRE, the authors suggested a correlation between the use of carbapenems and the high risk of acquisition. They also noticed that many other antibiotic classes had a similar correlation, including fluoroquinolones, third generation cephalosporins and others [59]. All these findings, with the paradoxical effect observed with certain other antibiotics, including vancomycin should lead us to reconsider the risk associated with different antibiotic classes [60,61].

### 3.4. Antibiotic Concentration in Gut Microbiota

The antibiotic concentration in the intestinal microbiota seems to be a major factor associated with the risk of MDRO acquisition. The variable diffusion of an antibiotic at the different digestive levels, duodenum or colon, will probably have an impact on the total concentrations affecting the microbiota.

For many years, the idea of digestive diffusion was the only factor associated with the risk of the emergence of resistance. Therefore, we could have wrongly supposed that molecules of the same antibiotic class could have a different impact on microbiota. Indeed, following the initial work of Hamzepour et al., we considered the difference in the impact of ceftriaxone (antibiotic with biliary elimination) and cefotaxime (antibiotic with urinary elimination) on intestinal microbiota [38].

Moreover, animal studies carried out in mice, to evaluate the role of antibiotics in the acquisition and persistence of ESBL-producing *Klebsiella pneumoniae* suggested that certain antibiotic classes, considered to have a high ecological effect, were able to inhibit colonization by Gram-negative pathogens. In this study, ertapenem suppressed the colonization by ESBL-producing *K. pneumoniae* strains, whereas imipenem-cilastatin did not promote or suppress colonization. These findings were explained by the high excreted concentrations of ertapenem in the intestinal tract, in contrast to the limited intestinal concentrations of imipenem-cilastatin that were incapable of altering the intestinal microflora [34,43].

In parallel, antibiotic concentrations in the intestinal tract, as an important factor associated with dysbiosis and the emergence of MDRO, have been confirmed in clinical studies. In a recent study dealing with the effect of imipenem on the intestinal microbiota’s composition, authors were surprised by an unexpectedly, high colonization rate with imipenem-susceptible ESBL-producing *E. coli*, before the antibiotic’s administration (70.6%), which remained stable despite imipenem-cilastatin administration. The authors suggested that the observed lack of impact could be explained by the known low biliary elimination of imipenem-cilastatin. Indeed, among carbapenems, there are significant differences in terms of biliary elimination [43,44]. Thus, it has become evident that the idea of biliary elimination alone is insufficient to explain the ecological impact of an antibiotic. In fact, considering the pharmacokinetics properties of an antibiotic together with its concentration at different levels of the bacterial microbiota, seems to be crucial for a better understanding of the matter.

More recently, two studies, the first conducted on healthy subjects and the other on hospitalized patients [39,40], demonstrated that although they have different elimination mechanisms, there was no difference between ceftriaxone and cefotaxime when used in clinical practice in regard to the emergence of MDRO or the modification of microbiota’s diversity and richness. This finding was explained by the fact that the total concentrations obtained, which corresponded to the doses administered and the elimination routes of these antibiotics, seemed to be equivalent for the two molecules.

However, certain broad-spectrum antibiotics with low intestinal concentrations, such as aminoglycosides and nitrofurantoin do not appear to have a significant impact on the intestinal microbiota. Thus, a study that included nine patients, colonized with ESBL-PE and treated with amikacin, showed no impact of amikacin on the intestinal microbiota [47]. Moreover, one study, conducted on a small population of women (*n* = 7) with recurrent urinary tract infections (UTIs) showed that nitrofurantoin did not affect Enterobacterales, *Enterococci* or yeast populations of intestinal microbiota during treatment. In this study, no resistant strains of Gram-negative aerobic bacteria were detected [62]. 

On the other hand, some antibiotic classes that have been identified as having little or no effect on intestinal microbiota compared to other classes, would have better results if we consider their pharmacokinetic characteristics. For example, orally administrated metronidazole is almost completely absorbed in the small intestine. Therefore, intestinal concentrations of metronidazole decrease significantly along the gastrointestinal tract, with minimal amounts reaching the colon. In contrast, poorly absorbed antibiotics, such as vancomycin, maintain high concentrations throughout the GI tract following oral administrations. This fact suggests that along the gastrointestinal tract, orally administrated metronidazole may have less effect on intestinal microbiota than oral vancomycin. Indeed, in a recent study, the authors demonstrated that oral vancomycin induces drastic and consistent changes in the human intestinal microbiota with a significant depletion of bacteria from the genus *Bacteroides* and an expansion of Enterobacterales, such as *E. coli* and *K. pneumoniae* [63].

Moreover, while the concentration of antibiotics in the gut seems to play a major role in the emergence of resistance, several recent studies also suggest that the impact of an antibiotic on the intestinal microbiota seems to be fast and occurs within the first administered doses. Thus, a study evaluating the impact of dicloxacillin, clindamycin, cefuroxime, cefotaxime and ciprofloxacin on the intestinal microbiota of mice showed a significant impact for clindamycin and dicloxacillin, which was observed after the second day of administration [64].

Additionally, there are many other factors involved in the concept of resistance to colonization [7]. For more than 50 years, it has been recognized that anaerobic bacteria of commensal flora provide protection against the acquisition of exogenous pathogens [58]. These factors have been widely studied in many previously published reviews. However, it is important to emphasize that the difference in composition between two microbiotas explains recent data suggesting inequalities in terms of bacterial antibiotic hydrolysis [65], clearance of acquired exogenous bacteria or MDRO [66]. Surprisingly, recent studies, measuring the effect of long-term antibiotic use on microbiota and the emergence of resistance, have shown no significant difference in the emergence of resistance, despite its effect on microbiota’s richness and diversity. Moreover, other studies have highlighted the important role of certain microbial species in the context of acquisition of resistant bacteria and/or the elimination of colonization by MDRO [32,67,68,69].

These findings help us to understand the effect of antibiotics, if they exist, on intestinal microbiota with regard to the emergence of antibiotic resistant bacteria. They also help us to understand the different impacts caused by different antibiotic classes on intestinal microbiota and to prioritize the use of those with less impact. Thus, human microbiota is diverse, dynamic and unequal when we consider the risk of resistance emergence. In a study evaluating the concentrations of ceftriaxone in the stool of five healthy volunteers, Leonard et al. highlighted the importance of endogenous beta-lactamase in the hydrolysis of ceftriaxone [65]. Thus, while all patients received the same doses of ceftriaxone, its residual concentration in the stool was variable, and even totally absent in some patients [65]. Furthermore, recent studies revealed that bacteria-producing beta lactamase inoculated with antibiotic-sensitive pathogens inhibited the efficacy of beta lactams [70]. Similarly, recent prevalence studies carried out in long-stay patients, showed significant differences between the composition of intestinal microbiota of patients colonized with ESBL-PE and non-colonized patients. More recently, in a study, carried out on travelers who acquired ESBL-PE during their stay abroad, the authors were interested in the speed of ESBL-PE decolonization [26]. They highlighted the difference in the composition of intestinal microbiota. Several other studies focusing on the acquisition and colonization with vancomycin-resistant *Enterococcus* (VRE), highlighted the importance of certain bacterial protective species. All these findings can largely explain the variable behavior of different microbiotas regarding the emergence of MDRO. Surprisingly, Keith et al., used an experimental mouse model to demonstrate the role of certain commensal bacteria in harboring resistance genes, in the degradation of antibiotics in the gut and the protection of intestinal microbiota from dysbiosis [69]. Indeed, in this model, the production of functional beta-lactamase by commensal *E. coli* strains, significantly reduced the clearance of these pathogens and enhanced their systemic dissemination during ampicillin treatment. The authors concluded that commensal bacteria, by acquiring resistance genes, can provide protection to exogenous pathogens from the bactericidal effects of antibiotics. 

## 4. In Hospitalized Patients, Other Factors Contribute to the Emergence of Resistance

Many authors have investigated the effect of different antibiotic classes on the emergence and acquisition of MDRO in hospitals. According to different studies, antibiotic therapy constitutes a necessary and crucial risk factor for MDRO acquisition; however, it is not the only factor. Indeed, published studies to date suggest that [58] if the overall antibiotic prescription is an important factor, the antibiotic class alone does not sufficiently explain the impact on intestinal microbiota. In fact, many studies comparing the effect of different antibiotic classes on the emergence of resistant bacteria suggest that this phenomenon does not depend on the antibiotic class alone. In a literature review on the antibiotic classes associated with the emergence of CPE, we were surprised to notice that carbapenems were not the only molecules involved, but that many other classes participated in the emergence of CPE [59]. This observation is also truer today in an era where multi-resistance is spreading within different microbial species [71]. Finally, some of these data explain the mixed and even disappointing results of studies focusing on antibiotic de-escalation, especially those considering reducing the antibiotic spectrum. Indeed, numerous studies [72,73] and a recent literature review [58] suggested that there was no reduction in the risk of emergence of MDRO when the antibiotic that was used had a reduced spectrum of activity, compared to maintaining an antibiotic with broad-spectrum activity. 

There are many other factors that contribute to the emergence of resistance. The inefficiency of antibiotic de-escalation policies to reduce the emergence of MDRO could be the result of not considering these various other factors. Among these factors, we must highlight the possible antibiotic effect of non-antibiotic treatments [74,75], the physiological modifications related to the patient’s clinical situation [76], and the consideration of the transmission of MDRO by hand.

Most of the published studies suffer from methodological limitations related to the microbiological methods used, the populations involved, and the lack of comprehensive reviews.

Besides the factors that have been forgotten and have not been considered in the various studies evaluating the ecological effects of antibiotics, the impact of other concomitant non-antibiotic treatments with antibiotic effects seems to be important. In fact, some commonly used non-antibiotic drugs have recently been associated with changes in the composition of the gut microbiota. In a study that analyzed more than 1000 marketed drugs and tested them on 40 intestinal bacterial strains, the authors found that 24% of the tested drugs, which belonged to different therapeutic classes, inhibited the growth of at least one bacterial strain in vitro [75]. Some classes, such as antipsychotics, were over-represented in this group [75]. A systematic literature review analyzing studies published in the last 20 years, reporting the antimicrobial activity of non-antibiotic drugs, highlighted 112 articles that explored the antimicrobial activity of non-antibiotic treatments [77]. Among these drugs, antidepressant, antihypertensive, anti-inflammatory, antineoplastic and hypoglycemic drugs demonstrated significant antimicrobial activity in vitro and in vivo against clinical isolates, Gram-negative, Gram-positive bacteria and fungi.

## 5. How Can We Apply These Findings in Clinical Practice

We have tried to demonstrate the complexity of the gut microbiota and its interaction with the administered antibiotics. Antibiotic prescription contributes to the emergence of resistance. Contrary to previously published theories, we believe that it is difficult to classify antibiotics according to their ecological effects.

In the hospital environment, antibiotic therapy must be considered as a trigger for resistance at the individual and collective levels. Indeed, many recent studies have underlined the role of antibiotic therapy in the emergence of MDRO in hospitals. This emergence encompasses two distinct but complementary phenomena: the selection of MDRO and its acquisition by hand transmission.

In fact, although many studies in intensive care units (ICU) [78,79] and outside ICU [80] have underlined the importance of antibiotic prescription in the acquisition of MDRO at the individual level, new studies have demonstrated the importance of the antibiotic prescription volumes [81] at the unit level in the acquisition of MDRO. Indeed, Harris et al. exposed 12 gut models to a pooled fecal slurry to CPE, before and after concomitant administration of piperacillin-tazobactam [82]. Before concomitant antibiotic exposure, the gut microbiota was disrupted, allowing the CPE’s proliferation. Also, if antibiotics are the triggers of resistance at the individual level, maintaining an antibiotic therapy provides a selective advantage to resistant bacteria and exposes a colonized individual to the proliferation of these species.

If we want to limit the spread of antibiotic resistance in the hospital environment, it seems important to reduce antibiotic consumption and to limit the transmission of MDRO by hand. Reducing antibiotic consumption requires a better definition of therapeutic indications, considering the need to introduce biomarkers into our clinical practice, and more importantly, a reduction in the duration of exposure to an antibiotic.

## 6. Conclusions

For twenty years, we have promoted and debated the concept of de-escalation considering that the ecological effect of antibiotics is molecular and class dependent. Even if this is still the case, we have no data that allows us to differentiate between different antibiotic classes in clinical practice. The complexity of the intestinal microbiota’s composition, the impact of the pharmacokinetic properties of antibiotics and the multiplicity of other factors involved in the acquisition and emergence of MDRO, lead us to think that de-escalation, especially since no study so far has shown its effectiveness, is not the solution to limiting the spread of MDRO.

More studies are needed to clarify the ecological risk caused by different antibiotic classes. In the meantime, it is important to concentrate our efforts on limiting antibiotic prescriptions to patients who really need it, and to work on reducing the duration of these treatments.

## Figures and Tables

**Table 1:** Impact of antibiotic on gut microbiota.

		Biliary Excretion	Activity on Anaerobic Bacteria		Induction of Dysbiosis	References
			Beta-Lactamase-Producing Anaerobic Gram-Negative Bacilli	Other Anaerobes		
Penicillin	Amoxicillin					[35]
	Amoxicillin-clavulanic acid					[35]
	Piperacillin-tazobactam					[36,37]
Cephalosporins	Ceftriaxone					[38,39,40]
	Cefotaxime					[38,39,40]
	Cefepime					[41]
Macrolides	Azithromycin					[42]
	Clindamycin					
Carbapenems	Imipenem				Inconclusive data	[43]
	Meropenem					[44]
	Ertapenem					[45]
Fosfomycin	Fosfomycin					[46]
Aminoglycosides	Gentamicin					[47]
	Amikacin					
Furanes	Nitrofurantoïn					[47]